# Editorial

**Published:** 1855-05

**Authors:** 


### THE MEDICAL EXAMINER.

### PHILADELPHIA, MAY, 1855.

A BILL FOR THE ESTABLISHMENT OF A BOARD OF MEDICAL CENSORS has lately been introduced into the Legislature of Pennsylvania. It contains a number of objectionable features, with which we think the profession throughout the State should be made acquainted. The bill provides for the establishment of a Board of Three Censors, to be appointed by the Governor, (and therefore, it is presumable, likely to be politicians,) who are to itinerate throughout the State, with authority to summon before them all practitioners of medicine, with a view to examination as to qualifications. Without a certificate from this Board no practitioner is hereafter to be allowed recourse at law to recover the amount of a bill for professional services, and a tax of *twenty-five dollars* is exacted for the certificate, which is to be available only for one year. An annual re-examination as to improvement in qualifications is also provided for by the bill, with an annual tax for certificate of *five dollars.*

It is scarcely possible, we trust, that so outrageous an interference with the rights of the profession in this Commonwealth can receive legislative sanction; but it will be well if those of our readers whose interests are affected by the scheme, will exert their influence to oppose it.

